# Clinical Pathology of the Blood

**Published:** 1902-03

**Authors:** 


					﻿Clinical Pathology of the Blood. A Treatise on the General
Principles and Special Applications of Hematology. By James
Ewing, M. D., Professor of Pathology in Cornell University
Medical College, New York City. In one handsome octavo vol-
ume of 432 pages, with 28 engravings and 14 full-page plates in
colors. Cloth, $3.50 net. Just ready. Lea Brothers & Co., Phil-
adelphia and New York. 1901.’
The author has endeavored to make this volume a practical
treatise on the subject by giving the clinical feature extensive
prominence. The temptation to make a theoretical book must have
been great, because the subject is strictly a laboratory one. But the
author has succeeded in producing a creditable work, for which
the profession will show due appreciation.
The first part of the book is devoted to a discussion of the inter-
pretation of blood analysis, giving its physiology and its pathology,
together with the technic, chemistry, morphology and physiology of
the red cells, the leucofcvtes and leucocytosis, the development of the
blood cells, the special pathology of the blood as in chlorosis,
progressive pernicious anemia, leukemia, pseudo-leukemia, in-
fantile anemia, splenectomy, etc.
In third part of the book is discussed the condition of the blood
in the acute infectious diseases, such as pneumonia, diphtheria, the
exanthemata, typhoid fever, syphilis, tuberculosis, leprosy, and
many other miscellaneous infective diseases.
The last part is devoted more directly to constitutional diseases,
including nervous and mental malignant diseases, and animal par-
asites as found in malaria, relapsing fever, etc.	T. J. B.
				

